# Minority MOMP: a toxic, slow demise

**DOI:** 10.18632/oncotarget.27753

**Published:** 2020-09-29

**Authors:** Yuan Xu, Deborah R. Surman, R. Taylor Ripley

**Keywords:** MOMP, Mcl-1, carcinogenesis

Thoracic cancers, including lung cancer, esophageal cancer, and mesothelioma, accounted for nearly 250,000 new diagnoses in 2018 [1]. The common link between these cancers is the chronic exposure to known environmental carcinogens such as cigarette smoke, bile reflux, and asbestos. Exposure to these carcinogens often occurs over decades and typically induces the development of greater than 3000 somatic mutations in the tumors [2, 3]. Therefore, a carcinogenic insult must repeatedly harm the cell enough to induce mutations while not strong enough to outright kill the cell. Minority MOMP is a mechanism of chronic, slow carcinogenesis that may explain the accumulation of mutations without cellular demise.

Resistance to apoptosis is a well-established hallmark of cancer [4]. Mitochondrial outer membrane permeabilization (MOMP) is the critical event that is mediated by the colocalization of Bax and Bak, membrane channel proteins, to the outer mitochondrial membrane (OMM). Toxic proteins such as cytochrome C (CytoC) are released from the mitochondrial inner membrane (MIM) space into the cytoplasm thereby activating the caspase system [5]. Following sub-lethal stresses, cells may survive when small percentages or a minority of mitochondria undergo MOMP [6]. This self-limiting MOMP is below the threshold necessary to trigger apoptosis—termed Minority MOMP. Caspase-mediated DNA damage occurs without cell death, which can explain the oncogenic transformation [7, 8]. MOMP is a switch-like event regulated by the balance of pro- and anti-apoptotic B-cell lymphoma 2 (Bcl-2) family members. While the activity of anti-apoptotic members such as Mcl-1, Bcl-2, and Bcl-xL raise the threshold for MOMP, the pro-apoptotic proteins such as BIM, BID, BAD, and PUMA reduce it. Environmental carcinogens may make cells withstand stressful stimuli and avoid apoptosis by upregulating anti-apoptotic proteins.

In our recent study published in Oncogene, we established an *in vitro* malignant transformation model to develop a systematic approach to test whether Minority MOMP was involved in bile acid-induced esophageal carcinogenesis [9]. In our prior work, we noted that cigarette smoke condensate (CSC) and bile acids altered the mitochondria function and increased the cancer cell’s malignant potential [10]. These findings suggested that mitochondrial alterations occur with environmental toxic exposures. Therefore, we developed a long-term exposure model in pre-cancerous esophageal cells (Barrett’s cells) which were cultured with the oncogenic bile acid, deoxycholic acid (DCA), for one year. The exposed cells developed an oncogenic phenotype (DCA-T) with clone formation, tumorigenesis in mouse flanks, and significant increases in migration and invasion. In the DCA-T model, we observed DNA damage and apoptotic pathway activation with upregulation of the effector proteins of MOMP (Bax and Bak). Normally, these findings are associated with cell death. Yet, no apoptosis or loss of viability occurred. Therefore, we explored how DCA activated the apoptosis pathway but did not induce cell death. Given that MOMP is associated with mitochondrial membrane depolarization, releases CytoC from the MIM space into the cytoplasm, and activates the caspase system, we queried whether these changes occurred in addition to upregulation of Bax and Bak. Remarkably, we found a decrease in mitochondrial membrane potential, a small increase in the cytosolic fraction of CytoC, and activation of a caspase-3 reporter despite lack of frank apoptosis. All these findings support the counterintuitive hypothesis that Minority MOMP facilitates cellular transformation and carcinogenesis.

Next, we queried whether Minority MOMP was associated with resistance to apoptosis. Given that the apoptotic machinery was activated, did this mechanism simultaneously enable resistance to prevent Minority MOMP from shifting to complete MOMP? We explored whether the known Bcl-2 proteins that are associated with resistance to apoptosis were upregulated. We found that Mcl-1 expression was significantly increased in DCA-T cells. Therefore, we knocked down Mcl-1 and noted decreased mitochondrial membrane potential, increased cytoC release, and an increase of caspase activation beyond the threshold in which the cells could survive. These results indicate that Mcl-1 is critical for maintenance of Minority MOMP and blocking MCL-1 could shift Minority MOMP to complete MOMP.

Mcl-1 acts as a switch that can convert Minority MOMP to complete MOMP. Therefore, targeting Mcl-1 may be a therapeutic strategy in combination with other anti-cancer drugs. Mcl-1 inhibition is known to be effective against several solid, cancer-derived cell lines [11]. To evaluate whether this strategy is feasible, we performed BH3 profiling, a functional live-cell assay, to evaluate the apoptotic threshold. The data revealed that cells with Mcl-1 inhibition were much closer to the apoptotic ‘threshold’, which suggests that Mcl-1 is critical to maintain the ‘unprimed’ mitochondria. Once the cell becomes ‘primed’ by targeting Mcl-1, that cell or tumor may become more susceptible to standard cytotoxic chemotherapy. The development of Mcl-1 inhibitors in human trials provides a translational potential by disrupting Minority MOMP through targeting of Mcl-1.

**Figure 1:**
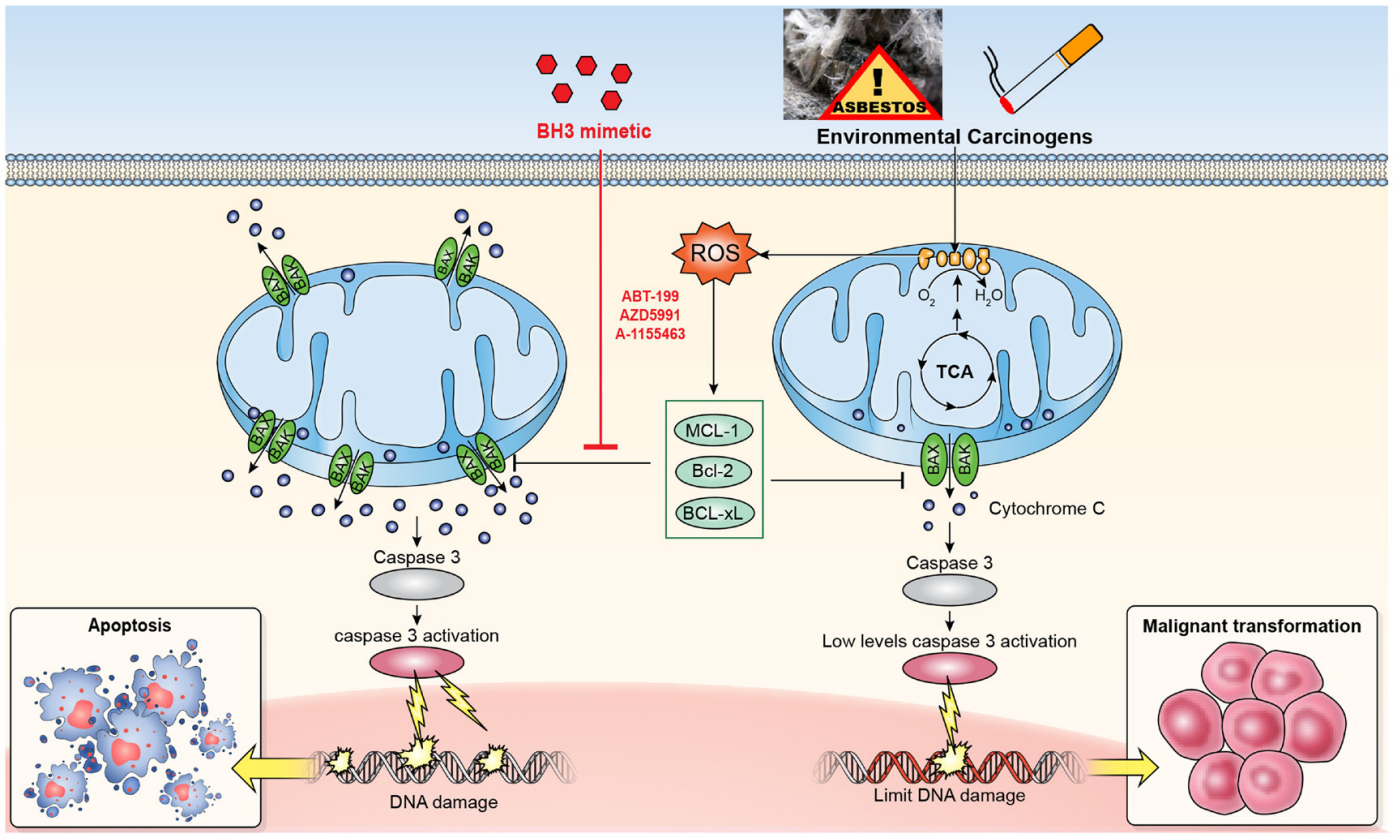
Proposed mechanism of minority MOMP promotes carcinogenesis induced by environmental carcinogens. While Minority MOMP promotes carcinogenesis, it may simultaneously upregulate anti-apoptotic proteins (MCL-1, Bcl-2, and BCL-XL) to protect the cancer cell from undergoing apoptosis. By directly targeting these proteins with BH3 mimetics, the reestablishment of cellular sensitivity to apoptosis through the disruption of Minority MOMP may overcome resistance in treatment-refractory cancers.

In summary, Minority MOMP is a newly discovered mechanism that may be common among cancers induced by environmental carcinogens. While Minority MOMP promotes carcinogenesis, it may simultaneously upregulate anti-apoptotic proteins to protect the cancer cell from undergoing apoptosis. By identifying and directly targeting these proteins, the reestablishment of cellular sensitivity to apoptosis through the disruption of Minority MOMP may overcome resistance in treatment-refractory cancers (Figure 1). Additionally, BH3 profiling is a novel bioassay that can measure the relative interactions of the apoptotic proteins to determine if specific therapeutics can shift mitochondrial threshold toward apoptosis. Future study will combine these highly translational potential bioassays for the development of precision medicine-based clinical trials.
